# Artificial intelligence in drug research and development: a review of methods and applications in drug repurposing

**DOI:** 10.1093/bib/bbag203

**Published:** 2026-05-21

**Authors:** Aleksandra Zielińska, Michał Fornalik, Mateusz Szczepaniak, Mariola Gimla, Anna Lemańska, Judyta Cielecka-Piontek, Eliana B Souto

**Affiliations:** Department of Pharmacology and Phytochemistry, Institute of Natural Fibres and Medicinal Plants – National Research Institute, Kolejowa 2, 62-064 Plewiska, Poland; Faculty of Medicine, Poznan University of Medical Sciences, Collegium Maius ul. Fredry 10, 61-701 Poznan, Poland; Oklahoma Medical Research Foundation, 825 NE 13th St.Oklahoma City, 73104 Oklahoma, United States; Oklahoma Medical Research Foundation, 825 NE 13th St.Oklahoma City, 73104 Oklahoma, United States; Oklahoma Medical Research Foundation, 825 NE 13th St.Oklahoma City, 73104 Oklahoma, United States; Department of Medical Biology and Genetics, Faculty of Biology, University of Gdansk, Wita Stwosza 59, 80-308 Gdansk, Poland; Faculty of Medicine, Poznan University of Medical Sciences, Collegium Maius ul. Fredry 10, 61-701 Poznan, Poland; Department of Pharmacology and Phytochemistry, Institute of Natural Fibres and Medicinal Plants – National Research Institute, Kolejowa 2, 62-064 Plewiska, Poland; Department of Pharmacognosy and Biomaterials, Poznan University of Medical Sciences, Rokietnicka 3, 60-806 Poznan, Poland; UCD School of Chemical and Bioprocess Engineering, University College Dublin, Belfield, Dublin 4, D04 V1W8 Dublin, Ireland

**Keywords:** artificial intelligence, drug repurposing, machine learning, deep learning, big data, pharmaceutical research and development

## Abstract

Artificial Intelligence (AI) plays an increasingly significant role in drug research and development, particularly in drug repurposing, which involves identifying new therapeutic indications for existing pharmacological compounds. From classical algorithms-based tools e.g. DrugRep and DrugRepo, to more recent innovations (e.g. RosettaVS, RepurposeDrugs), this paper reviews the latest AI methods (e.g. AlphaFold3, mediKanren, AdaDR, TxGNN) and their design applications in drug repurposing, particularly in machine learning, deep learning, and biological network analysis. It discusses strategies using predictive models, natural language processing, and big data analysis to accelerate the identification of promising candidates for clinical repurposing. Data availability, model interpretability, and results validation challenges are also highlighted. This review suggests that AI represents a groundbreaking tool in drug repurposing, ranging from protein structure prediction to knowledge graph reasoning, which can significantly reduce the time and costs of developing new therapies.

## Graphical Abstract

**Graphical Abstract:**
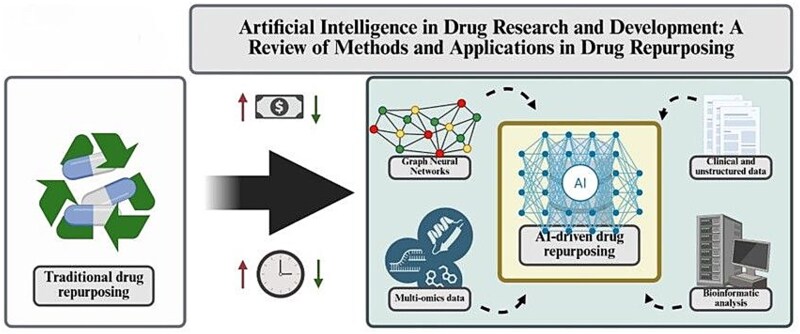

## Introduction

The pharmaceutical research landscape has the potential to be profoundly transformed, driven by integrating artificial intelligence (AI) technologies into drug discovery and development [1]. Traditional drug discovery is notoriously time-intensive and cost-prohibitive, often requiring over a decade and billions of dollars to bring a single compound to market. High attrition rates during clinical trials and the growing urgency to address unmet medical needs intensify these challenges [2].

Identifying new therapeutic indications for pharmacological agents offers a pragmatic solution to these inefficiencies. Drug repurposing, also termed repositioning or reprofiling, aims to systematically identify novel therapeutic applications for existing drugs. These may include approved medications, compounds investigated in clinical trials, or even those previously classified as failures, discontinued, or forgotten [3]. It is common for drugs to exhibit additional biological activities beyond their primary indications. Up to one-third of newly introduced compounds display at least one additional activity; however, opportunities to discover new uses for existing medications remain underexplored, limiting their true therapeutic potential [4].

This strategy accelerates development timelines and reduces research and development (R&D) expenditure by leveraging compounds with established safety profiles. Recent advances in AI, particularly in machine learning (ML), deep learning (DL), and natural language processing (NLP), have further empowered this approach by enabling the large-scale analysis of structured and unstructured biomedical data [5–7]. These technologies allow for analyzing large datasets and identifying patterns beyond traditional manual processing capabilities [1]. Beyond data analysis, AI can be implemented at every stage of the drug discovery pipeline, from target identification to clinical trials. Advanced molecular techniques, such as genome sequencing and proteomics, now provide insights into disease pathogenesis far more rapidly than in previous decades, but also generate large-scale datasets, whose understanding may be challenging. AI-facilitated analysis of these datasets helps pinpoint genes or pathways contributing to pathogenesis or treatment response in various diseases [8], leading to the identification of novel therapeutic targets or supplementary therapies [9, 10].

AI-driven methodologies can uncover latent relationships between drugs, targets, and diseases, facilitating the prediction of novel indications and improving success rates in early-phase trials. As such, AI is not merely increasing existing repurposing workflows, but it is reshaping the paradigm of therapeutic innovation [5, 6, 11].

Currently, introducing a new compound to the market requires significant resources, including financial investment, personnel, and years of research before Phase I clinical trials even begin. Estimates suggest that bringing a new drug to market takes 13–15 years and costs over US$2 billion, with these numbers gradually increasing [12]. Over the years, an inverse trend has emerged: despite greater funding allocated to R&D, the number of new compounds entering the market has declined [13]. Regulatory requirements and legislative processes for translating discoveries into clinical applications often hinder the development of novel therapies [14]. Drug repurposing addresses these challenges by reducing R&D costs by an estimated 40%–60%, as preclinical safety and toxicology studies for existing drugs have already been completed [15]. From an industrial perspective, repurposed drugs reach Phase II trials up to five years faster than novel compounds, with FDA approval rates potentially three times higher. Furthermore, drug repurposing offers a new response to unmet medical needs and drug shortages, with the market projected to grow 4%–15% annually over the next decade [16, 17]. Recent advances in AI technologies have further accelerated these developments, unlocking new opportunities for therapeutic discovery. This review discusses the intersection of AI and drug repurposing, highlighting methodological advancements, case applications, and future directions in this rapidly evolving field.

## Drug repurposing–Classical versus modern approaches

Historical drug repurposing strategies relied on three primary approaches: drug-centric, disease-centric, and target-centric methodologies [18]. Drug-centric approaches focus on expanding applications of existing pharmaceuticals by examining their pharmacological profiles, including known activities, off-target effects, off-label uses, failed trials, or expired-patent drugs. A well-known example is sildenafil (Viagra®), a selective inhibitor of cyclic guanosine monophosphate-specific phosphodiesterase type 5. Initially developed by Pfizer in the 1980s for hypertension and angina, sildenafil failed in clinical trials for cardiovascular indications. However, it’s unexpected side effect—improved erectile function—led to its successful repositioning for erectile dysfunction [19, 20]. Another example is thalidomide, initially marketed as a sedative but withdrawn due to teratogenic effects that led to thousands of congenital disabilities. Its antiangiogenic properties were later leveraged to repurpose it as an anticancer drug [21].

Disease-centric approaches shift the focus from the drug to the pathology to match compounds to diseases based on shared molecular signatures or pathophysiological pathways. For instance, azidothymidine, synthesized as a potential anticancer agent, was repurposed as the first human immunodeficiency virus (HIV) treatment after discovering that it can inhibit HIV reverse transcriptase, a key viral replication enzyme [22]. This case not only underscores the success of the disease-centric approach but also illustrates how repurposing, when guided by molecular insights, can rapidly transform patient care.

Target-centric approaches focus on repurposing compounds that modulate well-characterized molecular targets. Tamoxifen, developed initially as a post-coital contraceptive, was repurposed for breast cancer after its anti-estrogen activity was discovered [23, 24]. Another notable example is nitroxoline, a well-established antibiotic traditionally used as a first-line treatment for uncomplicated urinary tract infections due to its favorable pharmacokinetics, including prolonged urinary retention [25, 26]. Preclinical *in vitro* and *in vivo* studies have demonstrated its ability to inhibit angiogenesis [27], reduce cell viability and migration, and induce apoptosis in bladder cancer cells [28]. Furthermore, emerging *in vitro* evidence suggests that nitroxoline may inhibit replication of the mpox virus, highlighting its potential for repurposing as an antiviral agent [29].

Each traditional strategy has unique limitations. Drug- and disease-centric approaches may overlook disease-specific interactions, while target-centric methods require detailed structural and binding affinity data, complicating large-scale *in silico* screening. Although traditional approaches have yielded notable successes, the growing number of synthetic molecules, databases, and available clinical evidence poses a challenge. The breadth and heterogeneity of available data significantly impair effective integration, analysis, and interpretation [30]. The creation of databases is time-consuming and often obsolete by the time of publication due to the influx of new data [31]. The reliance on manual literature review and individual *in silico* analysis creates a substantial bottleneck in candidate identification. While *in vivo* and biochemical methods remain valuable for elucidating drug–target interactions, their high cost, tedious, and labor-intensive nature significantly hinders their applicability in large-scale drug repurposing, where efficiency is critical [32]. This issue is particularly pronounced in rare diseases, where limited clinical data further restrict the scope for discovery and validation [30, 33].

The principle behind AI-driven drug repurposing remains similar to traditional methods: it must be evidence-based and data-driven. The data used to identify new applications for existing molecules can be categorized as unstructured versus structured. Unstructured biomedical data account for approximately 80% of all medical data [34]. While lacking standardized definitions, this category includes textual and imaging data from clinical practice and biomedical scientific publications [35]. Research articles, for example, provide unstructured information that can be integrated into drug repurposing pipelines using NLP, particularly with transformer models.

Transformer models are DL architectures based on understanding words through contextual relationships via self-attention, and have revolutionized NLP [36]. Self-attention mechanisms analyze the relationships between different positions in a sequence to generate contextualized data representations. Since the introduction of transformer architectures, numerous large language models (LLMs) have emerged, including BERT, RoBERTa, ELECTRA, and the widely recognized GPT series (later commercialized as ChatGPT) [37–40]. These models are pre-trained on vast amounts of textual data, requiring only fine-tuning rather than complete training for specialized tasks. However, biomedical research involves domain-specific terminology and linguistic patterns distinct from general language. Specialized models like BioBERT, SciBERT, PubMedBERT, and BioELECTRA outperform general-purpose LLMs’ accuracy and reliability for biomedical applications [41, 42].

These NLP models have proven particularly effective for extracting drug-target interactions from scientific articles, enabling standalone drug repurposing or integration into broader pipelines for analyzing unstructured data. This approach offers a critical advantage over curated interaction databases: it allows researchers to explore over 30 million PubMed articles, uncovering novel associations [43].

**Figure 1:**
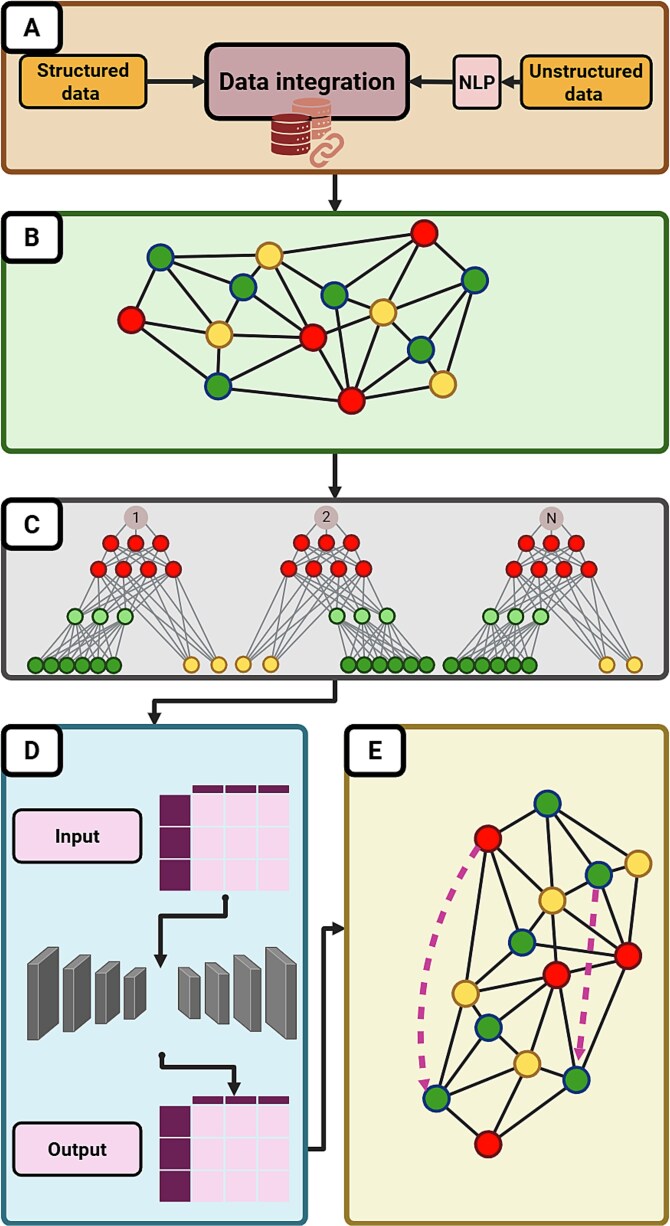
Pipeline for graph-based drug repurposing: [A] Data integration: A workflow begins with a comprehensive data integration, which ideally should include both structured data (e.g. DrugBank, ChEMBL, STRING, DisGeNET) and unstructured data sources (e.g. literature, clinical notes processed via natural-language processing). [B] Data harmonization: The data should be harmonized through entity normalization and identity mapping. [C] Knowledge graph construction: These data are incorporated into a knowledge graph with nodes for drugs/proteins/genes/diseases and links between them. [D] Embedding stage: In the embedding stage, the complete multidimensional graph is projected into a low-dimensional vector space using methods such as node2vec, GraphSAGE, or an alternative. This captures local environmental structure and note characteristics (e.g. chemical fingerprints, sequence features). [E] Machine learning implementation: Machine learning models are implemented on these embeddings. [F] Link prediction and scoring: Link prediction algorithms score and rank drug-target associations.

State-of-the-art NLP models achieve >99% accuracy in identifying articles documenting drug-target interactions [44]. Furthermore, automated text-mining tools like PubTator, BEST, and GDPminer act as AI-powered biocurators, systematically extracting and organizing entity-specific information (e.g. genes, drugs, diseases) from literature [45–47]. After NLP processing, unstructured data can be combined with structured data, which commonly involves curated databases, such as ChEMBL, BindingDB, PubChem, GtopDB, DrugBank, GNBR, or DrugTargetCommons [48–53], to create holistic, multi-modal frameworks for drug repurposing. Figure 1 depicts the workflow of graph-based drug repurposing. Table 1 shows a comparative analysis between traditional and AI-based methods.

**Table 1:** Comparative analysis between the traditional methods and AI-based methods.

Aspect	Traditional methods	AI-based methods
Advantages	Leverage existing clinical and pharmacological data, established human safety and pharmacokinetic data (e.g. prior trials), which accelerate clinical deployment and lower risk, proven track record with many successful therapies across diverse diseases; Generally high interpretability based on known drug mechanisms and clinical experience.	Data-driven discovery: AI mines vast and diverse biomedical datasets to uncover non-obvious drug-target links, which likely would be overlooked otherwise, and high-throughput *in silico* screening can rapidly prioritize candidates.
Drawbacks and Limitations	Limited by existing knowledge, Incomplete data on drug effects can bias discovery, and still require full efficacy trials.	Relies on large, high-quality datasets; biased data may lead to false-positive results. Often, a ‘black box’ lacks justification for predicted associations; however, newer models start to implement mechanistic explanations to improve interpretability.
Types of Data Used	Pharmacological and clinical data, biomedical literature	Broad, multi-modal data: integrate chemical and structural libraries, genomics, proteomics and transcriptomics, electronic health records, side-effect/adverse-event databases, and biomedical text. Can employ knowledge graphs and combine heterogeneous datasets.
Methodological Approach	Hypothesis-driven and mechanistic: drug-centric (testing a new indication for a known drug), disease-centric (screening existing drugs for a disease with known pathophysiology), or target-centric (matching drugs to a biological target)	Data-driven approach: employs ML, network analysis, and NLP. Examples include graph neural networks on biomedical knowledge graphs and deep understanding of chemical-target interaction networks.
Scalability	Low throughput and labor-intensive: often requires bench experiments and focused screenings.	High scalability, as AI methods can screen millions of compounds or data points in parallel. Computational predictions still require rigorous experimental and clinical validation.

## Biological network analysis and graph-based methods

All the extracted data can be incorporated into a Knowledge Graph (KG) or other forms of biological network analysis. These frameworks map biological systems—proteins, genes, diseases, or drugs—as interconnected nodes, with edges representing interactions between them. Traditionally, the importance of a node (e.g. a protein) to an organism or disease depends on the number of its connections [54]. For example, a protein with many interactions in a protein–protein interaction (PPI) network may play a central role in disease mechanisms. Network modelling aligns with the three classical drug repurposing strategies: drug-centric, disease-centric, and target-centric approaches [55]. Edges in these networks can be unidirectional (showing non-hierarchical associations) or directional (indicating causality, regulation, or activation). Graphs may include one or both types of connections [56]. Researchers can identify drugs with shared mechanisms by creating association networks and applying topological measures like betweenness centrality (identifying nodes that act as bridges), closeness (measuring how easily a node interacts with others), or discovering diseases with overlapping pathways [55]. However, in addition to known connections, this method is facilitated by ML, which recognizes patterns most likely unrecognizable to humans.

Before applying ML algorithms to predict novel associations in a graph, the structural information (nodes and edges) must be transformed into fixed-length numerical vectors—a process called graph embedding [57]. Embeddings preserve the direct connections (e.g. interactions between nodes) and the broader graph structure (e.g. a node’s role or position). This is achieved through two key principles: (i) Contextual Representation captures a node’s immediate neighbourhood and its direct connection; (ii) Structural Equivalence maps nodes with similar roles or positions in the graph (e.g. proteins acting as hubs in a network). Embeddings enable ML models to process graph data, which inherently lacks the fixed-size input format most algorithms require. The choice of embedding method depends on the graph’s composition. Few of the exemplary techniques include: (i) node2vec, which generates vector representations for nodes by simulating random walks over the graph; (ii) FastRP, which aggregates information from neighboring nodes at varying distances and applies random linear projections to compress features into lower-dimensional vectors; and (iii) GraphSAGE, which utilizes local node information (for example chemical properties) and use the information of closeby nodes to update the first node vector [57–59]. In practice, hybrid approaches combining multiple methods are also implemented, leveraging complementary strengths.

**Figure 2:**
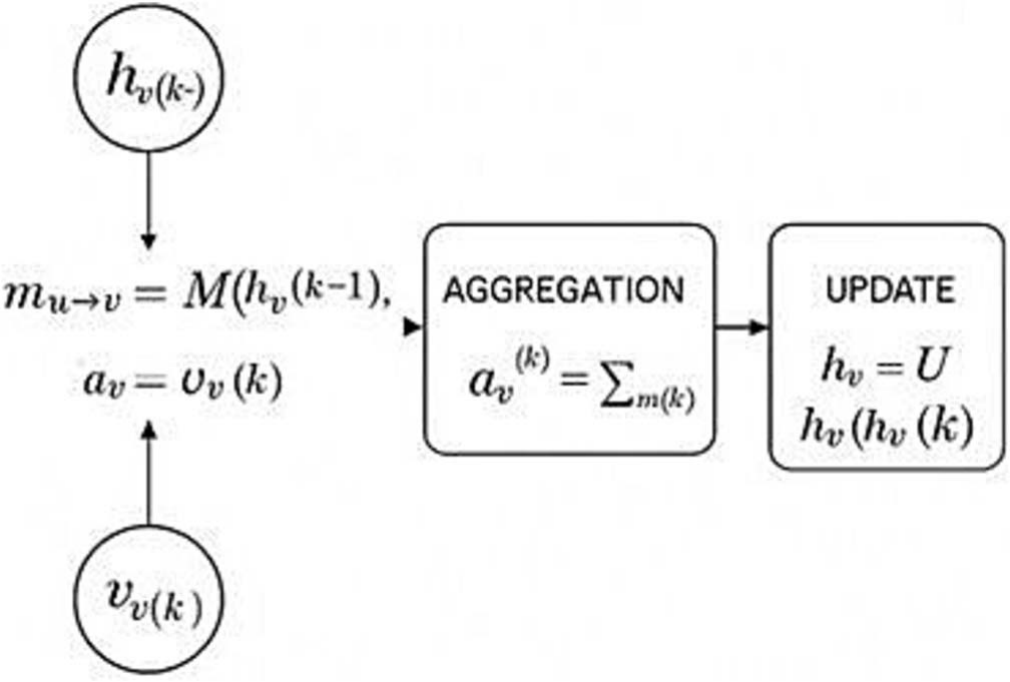
Message-Passing Layer in a Graph Neural Network.

Graph Neural Networks (GNNs) are ML frameworks designed to analyze and learn from graph-structured data. GNNs operate through iterative message-passing mechanisms—each node aggregates information from its immediate neighbors. By stacking multiple layers, GNNs hierarchically capture local molecular properties and global topological features, enabling GNNs to predict novel drug-target interactions [60]. The mechanism behind message-passing layers is presented in Fig. 2.

At the ${k}_{th}$ iteration, the embedding of node $v$ is updated by the following steps:

1. Message computation ${m}_{u\to v}^{(k)}=M\left({h}_u^{\left(k-1\right)},{h}_v^{\left(k-1\right)},{e}_{uv}\right)$ (where ${h_u}^{\left(k-1\right)}$, ${h_v}^{\left(k-1\right)}$ are the embeddings of nodes $u$and $v$ from the layer $k-1$, ${e}_{uv}$ is the feature vector of edge $u,v$, $M$ is the message function)
2. Aggregation $${a}_v^{(k)}={\displaystyle \begin{array}{c}\oplus \\{}u\in \mathcal{N}(v)\end{array}}\ {m}_{u\to v}^{(k)}$$ (where $N(v)$ is the set of neighbors of $v$, ⨁ is a permutation-invariant aggregator e.g. mean, sum, max)
3. Update ${h}_v^{(k)}=U\left({h}_v^{\left(k-1\right)},{a}_v^{(k)}\right)$ (where $U$ is the update function, ${h_v}^{(0)}={x}_v$ is the initial feature vector of node $v$)

A single GNN layer relies on a parametric, permutation-equivariant mapping that updates each node’s representation and is optimized during training. The message-passing mechanism operates in three elementary steps. First, for every edge $\left(u,v\right)$, a message is computed using the embeddings of nodes $u$ and $v$ from the previous layer *h_u_^(k-1)^* and *h_v_^(k-1)^*, along with edge-specific features (e_uv_). ${m}_{u\to v}^{(k)}=M\left({h}_u^{\left(k-1\right)},{h}_v^{\left(k-1\right)}{e}_{uv}\right)$. Second, these messages are combined into a single aggregated value using a permutation-invariant operator ⊕ (e.g. summation, mean, or maximum) $${a}_v^{(k)}={\displaystyle \begin{array}{c}\oplus \\{}u\in \mathcal{N}(v)\end{array}}\ {m}_{u\to v}^{(k)}$$, which ensures the result remains unchanged regardless of the order in which neighboring nodes are processed. Third, the node’s updated embedding h_v_^(k)^ is generated by applying a learnable function *U* to the aggregated message and its previous state. Because both $M$ and $U$ are shared across the graph and $\oplus$ is insensitive to neighbor ordering, the entire layer commutes with any relabeling of nodes, ensuring that GNNs respect the intrinsic symmetries of graph-structured data. By stacking $K$ such layers, each node’s final embedding ${h_v}^{(K)}$captures information from its $K- hop$ neighbourhood, i.e. local topology, node features, and edge attributes are fused in a fully differentiable pipeline.

Recent advances in GNNs have been tailored for drug repurposing by embedding large-scale biomedical networks and predicting novel drug-disease associations. These models combine breadth (analyzing thousands of compounds) with depth (uncovering mechanistic insights) by mining complex biomedical graphs. For example, deepDR integrates ten interlinked networks—including drugs, diseases, drug targets, and side effects—using a multimodal autoencoder to learn unified pattern embeddings. This framework clusters related entities in the graph, enabling systematic discovery of repurposing candidates [61]. Other GNN pipelines, such as GDRnet, encode molecular graphs into a four-layered heterogeneous network comprising drugs, diseases, genes, and anatomical structures. GDRnet treats drug-target interaction prediction as a link-prediction task. Its encoder-decoder architecture is trained end-to-end to score drug-disease pairs directly, bypassing traditional embedding-and-ranking workflows [62]. VirtuDockDL employs GNNs to characterize compound-target interactions, optimizing efficiency in large-scale virtual screening. Learning spatial and chemical features of molecular complexes prioritizes high-affinity binding candidates for experimental validation. These pipelines are often built on publicly available datasets that incorporate and merge diverse data from public repositories [63]. One of the most notable examples is the Drug Repurposing Knowledge Graph (DRKG). This structured biomedical dataset maps relationships between genes, chemical compounds, diseases, biological processes, drug side effects, and symptoms. DRKG provides a unified platform to uncover therapeutic opportunities that might otherwise remain hidden. For example, DL models applied to the DRKG identified candidate drugs for repurposing against SARS-CoV-2 by analyzing shared molecular pathways between existing compounds and viral mechanisms [64]. Similarly, network clustering within the DRKG highlighted compounds targeting neuroinflammatory or protein aggregation pathways relevant to amyotrophic lateral sclerosis pathology [65]. Additionally, DRKG can be modified to focus on a specific subset of diseases, including RDK-115 – a version tailored for rare diseases. This analysis revealed that 438 of 870 predicted drug-disease pairs (50.3%) were novel, with 43.2% ranked as high-confidence candidates. Of these, 48.3% recapitulated known associations, validating the model’s accuracy. Moreover, among small-molecule-disease pairs, 44 of 62 predictions (71.0%) were newly inferred, with 31.8% prioritized as top-tier leads [66].

Recent advancements in GNNs for drug repurposing include tools, like AdaDR, which employs an adaptive graph convolutional network to deeply integrate node features (e.g. drug chemical properties, direct connections) with global topological dependencies in biomedical networks [67]. AdaDR operates through three sequential steps: (i) a graph convolution module that embeds drug and disease information into low-dimensional vectors; (ii) an adaptive learning module that uses attention mechanisms to weigh the importance of node connections; and (iii) a prediction module that concatenates these embeddings to infer novel drug-disease associations [67].

Another innovative framework, TxGNN, combines GNNs with metric learning to enable zero-shot drug repurposing—a method designed for scenarios with minimal labeled data, such as rare diseases where up to 95% lack approved therapies [67, 68]. TxGNN was validated across over 17,000 diseases, including those without existing treatments. While the GNN component learns relationships between drugs and diseases, the metric learning module calculates similarity scores to prioritize associations. A key advantage of TxGNN is its human-centric interpretability, which simplifies clinical translation by providing intuitive explanations of predictions, even for users with limited computational expertise.

A partially analogous approach, case-based reasoning over subgraphs (CBR-SUBG), uses GNNs to recognize patterns in biomedical graphs and combines them with query-specific subgraphs to predict repurposing candidates [69]. CBR-SUBG identifies drugs with similar network neighborhoods and infers their therapeutic potential based on known analogous interactions. For example, it successfully repurposed propranolol (an antihypertensive drug) for Parkinson’s disease due to its anti-tremor effects, mirroring its approved use [70]. CBR-SUBG outperforms classical GNN-based link prediction models by emphasizing mechanistic explainability—it constructs subgraphs of similar drugs and diseases to contextualize predictions biologically. However, in each case of prediction of therapeutic potential, a clinical evaluation remains essential. For example, meta-analyses associate propranolol with increased Parkinson’s disease risk, which could have resulted from prescribing this drug for the disease-related tremor, therefore not indicating the causality [71]. Furthermore, newer studies suggest that it may reduce tremor-related cortical activity [72]. Thus, each prediction should be experimentally or clinically evaluated.

## Structural modelling and virtual screening of compounds

Structural modelling methods aim to elucidate protein functions and their interactions with ligands, a critical step in identifying drug targets and evaluating potential compounds. These methods rely on predicting the three-dimensional (3D) conformations of proteins, including both active binding sites and allosteric regulatory regions. Modern virtual screening leverages AI to predict the activity of potential drug candidates by aligning chemical structures with target proteins, enabling rapid evaluation of vast chemical libraries [73]. Automated platforms, such as DrugRep, streamline repurposing workflows by integrating binding-pocket detection, molecular docking, and similarity-based ranking into end-to-end pipelines [74]. Similarly, other tools like DrugRepo aim to combine diverse approaches, that integrate similarities between known and candidate drugs based on their chemical structure, shared genetic associations with diseases, and protein–protein interaction networks [75]. However, DrugRep and DrugRepo rely on classical algorithms, whereas emerging AI-driven approaches employ DL to refine binding affinity predictions beyond traditional force-field methods.

For example, RosettaVS enhances the Rosetta framework with neural networks to improve pose and affinity prediction accuracy [76]. Another tool called RepurposeDrugs is a user-friendly web app that employs a ML model trained on a curated dataset of approved and failed clinical trials. This enables predicting the probability of approval for specific drug-disease indications, making it accessible even to researchers without strong programming skills [77]. An additional next-generation drug library and information resource has also been proposed [78]. Recent innovations in AI-powered virtual screening are presented in Table 2.

**Table 2:** Recent innovations in AI-powered virtual screening.

AI tool	Features	Reference
DiffDock	A conditional diffusion model that generates ligand poses on protein surfaces with high selectivity, outperforming classical docking tools	[79]
Deep Docking	Combines ligand-based machine learning predictors with iterative docking cycles, accelerating ultra-large virtual screens by up to 100-fold for billion-compound libraries	[80]
DeepVS	A convolutional neural network that extracts atom- and residue-level features from docking outputs to enhance enrichment factors	[81]
Machine Learning Scoring Functions	Supervised learning and Quantitative Structure–Activity Relationship models refine classical force-field methods for precise affinity estimation.	[82]
DeepLPI	A multimodal framework that predicts interactions using protein sequences and SMILES strings - text-based representations of chemical structures	[83]
CNN-Based Virtual Screening	Uses 3D convolutional architectures to directly score protein-ligand complexes, surpassing classical benchmarks like AutoDock Vina and Dock	[84]

## Structure-based drug repurposing

Historically, computational drug discovery faced limitations due to incomplete structural data. For example, only ~27% of human proteins had experimentally resolved structures in the Protein Data Bank [85]. However, a significant milestone in structural biology was the release of AlphaFold by DeepMind, which provided predictions of structures of nearly all known proteins, significantly increasing the datasets feasible for analysis [83–88]. The software gains more complexity with each subsequent update, allowing us to draw more definitive conclusions. While its predecessor, AlphaFold2, accurately predicted static 3D structures of single-chain proteins from amino acid sequences, AlphaFold3 introduces a paradigm shift. Its multimodal framework models protein-ligand complexes, protein-nucleic acid interactions, and multi-protein assemblies within unified structural predictions. Structure-Based Drug Repurposing (SBDR) integrates molecular docking, virtual screening, and AI-driven structural predictions to identify novel therapeutic indications [89]. AlphaFold3, for instance, performs innovative modelling for proteins up to 500 residues long, achieving over 70% accuracy in benchmark test cases [90, 91]. This precision allows researchers to repurpose short therapeutic peptides (8–30 amino acids) by predicting detailed binding sites and modelling RNA interactions within dynamic biomolecular systems. By reducing false positives in virtual screening workflows, SBDR identifies new therapeutic uses for existing peptide drugs—particularly in oncology (e.g. targeting cancer metabolism in breast and colorectal models [92]), immunotherapy (e.g. repurposing peptides to block immune checkpoints or reduce inflammation [93]), and neurodegenerative diseases.

This capability makes AlphaFold3 uniquely powerful for SBDR. It should be noted that, while AlphaFol3 can currently generate structural models of protein-ligand complexes, these represent more of a geometric hypothesis rather than validated binding affinity. The latter still remains incompletely reliable, even when structural prediction is accurate [94, 95]. Predicted structures so far represent typically static conformations, whereas actual proteins remain in dynamic state. Therefore, conformational changes, induced fit-binding or allosteric effects may not be accurately represented by current models [96]. Member proteins remain challenging to predict their structure as historically, relatively small number of already structured proteins have been available to train the models [96]. Therefore, additional wet-lab biophysical assays still remain essential for experimental confirmation [95, 96].

Nevertheless, by demonstrating the binding affinity to the target structure by both methods, the therapeutic potential of the molecule in a new disease can be predicted. As AI advances, these computational strategies are transforming drug repurposing from a serendipity-driven process into a structured, data-driven approach, accelerating responses to urgent medical needs [97].

## Integration of big data and large-scale datasets

In drug repurposing, combining large-scale datasets, ranging from multi-omics profiles to chemical libraries and clinical records, enables AI models to uncover novel drug-disease associations. Integrative DL strategies fuse transcriptomic, proteomic, and metabolomic features into unified frameworks [98]. However, challenges arise from petabyte-scale data volumes, high-dimensionality, and privacy constraints, which impose heavy demands on computing and memory efficiency [99]. Training and running DNN on multi-omics matrices or simulating billions of compound-target interactions requires high-performance computing or hundreds of graphic processing units (GPUs), often generating costs for institutions in hardware and energy exceeding millions of dollars [100]. For example, one of the largest supercomputers in pharmaceutical industry, BioHive-2, owned by Recursion Pharmaceuticals, comprises 504 GPUs with a price of approximately 25–30 k USD per unit. This cluster represents roughly 15 million USD in hardware cost alone, before even accounting for energy infrastructure and usage, storage or the costs of the network [101]. Additionally, while computational power of supercomputers doubles now every nine months, with each passing years also doubles the cost of hardware and the energy [102]. Moreover, even with such infrastructure, petabyte-scale feature matrices can overwhelm node memory and saturate disk I/O, turning analyses that should take hours into multi-day processes. To address these limitations, deep autoencoders compress high-dimensional chemical and omics data into compact, lower-dimensional embeddings, preserving signal while reducing size [103]. Meanwhile, atomic-resolution repositories—like the Protein Data Bank (PDB), now exceeding 234 785 experimentally resolved 3D structures—provide rich molecular datasets but demand optimized preprocessing pipelines to handle their complexity [104].

## Applications of AI in drug repurposing—a review of studies and cases

AI’s leverage in drug repurposing has been demonstrated across multiple applications. One area where AI provides significant advantages—particularly in accelerating timelines—is emergency response scenarios, as exemplified by the COVID-19 pandemic. In a study by Messina *et al*. (2020) [105], an AI-driven network analysis using the Random Walk with Restart (RWR) algorithm identified host genes potentially implicated in SARS-CoV-2 pathogenesis. While RWR itself is not strictly an AI method, it is frequently integrated into broader AI workflows for drug repurposing. This approach leverages the guilt-by-association principle, which posits that proteins, genes, or nodes closely connected in biological networks are functionally related [106]. Applied to COVID-19, the analysis highlighted genes linked to DNA repair pathways and early immune activation, offering mechanistic insights into viral-host interactions and potential therapeutic targets [105].

Network-based DL frameworks and knowledge graphs also have proven useful in providing rapid insights into possible treatments. CoV-KGE was a knowledge graph developed by Zeng et al. (2020) [107] that includes 15 million edges across 39 relationship types: drugs, diseases, genes, pathways, and expressions, derived from 24 million PubMed publications. Validated against COVID-19 datasets, CoV-KGE achieved high predictive accuracy (AUROC = 0.85), identifying 41 repurposable drugs, including dexamethasone, indomethacin, niclosamide, and toremifene. Subsequent gene expression and proteomic enrichment analyses in SARS-CoV-2-infected cells corroborated these findings. Similarly, AI-driven network diffusion and proximity algorithms were utilized to screen more than six thousand drugs with anti-SARS-CoV-2 potential, revealing 918 possible candidates [108]. It was noticed that most algorithms offer predictive power for these factual data, but no single method provides reliable results in all data sets and metrics. This resulted in the development of a multimodal approach that combines the predictions of all algorithms. The proposed model allowed for the discovery that 76 out of 77 drugs that effectively reduced viral infection do not bind to SARS-CoV-2 target proteins, which indicates that these drugs rely on network-based actions that cannot be identified using docking-based strategies [108].

By analyzing network-based representations of disease-specific transcriptomic data, AI-driven solutions like the Genome-wide Positioning Systems (GPSnet) algorithm leverage mechanistic insights to identify genome-derived therapies. GPSnet repurposes drugs by mapping patient-derived DNA/RNA sequencing data onto protein–protein interaction networks, constructing disease-specific modules. This approach predicted drug-disease associations for 140 approved drugs, highlighting compounds, such as enzastaurin, linifanib, olaparib, pictilisib, refametinib, selumetinib, and vemurafenib, as promising candidates for breast, lung, and skin cancers. These predictions were experimentally validated *in vitro*, demonstrating efficacy in cell models [109].

The network technology was also used to virtually recreate a brain in the NEUBOrg, an AI-driven platform simulating whole-brain organoids (WBO), i.e. artificially induced brain models. It leverages the DeepNEU (v6.2) DL framework to model stem cell differentiation, cognitive maps, and neural network dynamics using recurrent neural networks, support vector machines, and evolutionary algorithms. This platform was applied to identify novel therapeutic strategies for metachromatic leukodystrophy (MLD), a rare neurodegenerative disorder. Using artificially generated whole-brain simulations (aiWBO), NEUBOrg created an aiWBO-MLD model replicating human brain organoids with arylsulfatase A (ARSA) deficiency—the genetic hallmark of MLD. The platform screened 861 drug combinations within this simulated environment, prioritizing 12 two-drug regimens that alleviated MLD-associated pathology. Top candidates included: (i) Lenvatinib-based combinations: Lenvatinib paired with pembrolizumab, rapamycin, regorafenib, sunitinib, or capmatinib, and (ii) Olaparib-based combinations: Olaparib combined with abemaciclib, palbociclib, regorafenib, ribociclib, or sunitinib, and other parings. These combinations reduced MLD symptoms *in silico*, demonstrating NEUBOrg’s ability to accelerate drug discovery for rare diseases with limited preclinical models [110].

Rare diseases represent a critical yet underexplored frontier for AI-driven drug repurposing. While they currently account for less than 5% of all AI-based repurposing efforts, early studies demonstrate promising potential [30]. For example, researchers applied ML to identify therapies for Pitt–Hopkins syndrome (PTHS), a rare genetic disorder [109]. By combining preclinical data from animal models with high-throughput screening of ion channels implicated in the disease, the calcium channel blocker nicardipine—commonly used for hypertension and angina [111]—was found to modulate dysfunctional ion channels in PTHS, suggesting therapeutic utility [109, 112].

Another study utilized a two-stage AI approach involving prediction and machine learning to analyze gene expression data from 262 patients with 31 diseases and 268 healthy controls. Tested diseases encompassed inclusion body myositis, polymyositis, and dermatomyositis. After clustering the diseases by gene expression, the efficacy of known small molecules was tested using the reversibility of abnormal gene expression. Using a tool that allows for establishing drug targets based on mimicking or reversing observed gene expression changes, 10 drugs corresponding to inclusion body myositis and polymyositis (alvocidib, gemcitabine, wortmannin, tosedostat) and 10 drugs corresponding to polymyositis and dermatomyositis (perhexiline maleate, selumetinib, viscostatin) and 14 drugs for all diseases, including reserpine, salermide, wortmannin, were identified [98].

For sarcoidosis and Wilms’ tumor, a network-based method leveraging the Global Network of Biomedical Relations (GNBR) prioritized 30 repurposing candidates. The top candidates, trifluoperazine (an antipsychotic repurposed for Wilms’ tumor) and everolimus (an mTOR inhibitor used in sarcoidosis), were proposed for clinical trials due to their mechanistic alignment with disease etiology [113].

Retinitis pigmentosa, a genetic disorder causing progressive blindness with no current therapies, was also targeted using the ML approach [114]. By integrating transcriptomic data, drug repositioning datasets, and mechanistic maps of retinitis pigmentosa, researchers identified 109 approved drug targets with potential therapeutic utility. This framework highlights ML’s ability to repurpose existing drugs for conditions lacking treatment options [115].

ML was also implemented in Noonan syndrome or LEOPARD syndrome. These autosomal disorders are manifested by short stature, facial dysmorphia, and cardiomyopathy [116]. ML analyzed genomic and drug interaction data to predict the efficiency of selected anti-cancer drugs, which might be useful in those conditions. Dasatinib—a leukemia treatment—emerged as a candidate for Noonan syndrome due to its inhibitory effects on dysregulated signaling pathways [117].

URSAHD (Unveiling RNA Sample Annotation for Human Diseases) is a ML tool that identifies disease-specific molecular features by linking gene expression profiles to clinical phenotypes. URSAHD validated cisplatin for refractory anemia with excess blasts and resveratrol for sideroblastic anemia [118].

Additionally, AI-driven methods can be utilized to identify not only a single drug candidate for repurposing but also combinations of drugs that, taken together, might present additional efficiency. Li *et al*. (2025) [119], using the DeepDrug repurposing framework, identified a combination of five drugs as a potential treatment for Alzheimer’s disease: tofacitinib, niraparib, baricitinib, empagliflozin, and doxercalciferol. The graph-based method was applied, and the results were incorporated into graph neural networks, which were further quantified into drug-gene scores for experimental validation [119]. A similar approach was used by Pan *et al*. (2023) [120], where they combined GCN and fully connected networks (FCN) to identify promising drug combinations for Alzheimer’s [120]. The GCN first learned the features from the drug-target interactions network, while FCN then predicted effective combinations.

Utilizing AI-based solutions can also help clinicians to predict the response to certain treatments and possibly mitigate the adverse effects. For example, common side effects of anthracyclines—a chemotherapeutic agent commonly used in pediatric cancer therapies—is anthracycline-induced cardiotoxicity (ACT) [121]. Before implementing AI solutions, predicting which patients will develop ACT was not possible. Fortunately, one study demonstrated that by executing classical random forest (RF) algorithms on either clinical factors, like patients’ characteristics and their therapy history, genetic factors, or their combinations, successfully predicted the ACT. The combined RF model was observed to outperform the clinical and genetic RF models with higher area under the curve (0.71), higher specificity, higher positive predictive value, and lower misclassification rate [122].

Similarly, as we transition into the era of personalized medicine, AI solutions can be integrated to forecast drug responses or stratify patients into populations that will derive the greatest benefit from a proposed therapeutic intervention [8]. By analyzing large multimodal datasets, AI-based approaches have successfully identified novel biomarkers across multiple disciplines, like neurology or oncology [123, 124]. For instance, an endophenotype-based network model derived from multi-omics and interactome data revealed that sildenafil may be a potent drug in Alzheimer’s disease in men, importantly with a simultaneously proposed mechanism [125]. In addition to identifying a single treatment target or selecting a most promising compound to target it, AI tools can be beneficial in diseases that may exhibit varying etiologies or mechanisms across different patient groups or disease stages, situations where ‘one fits all’ solutions are ineffective [126]. Combinatorial analyses incorporate various combinations of patients’ features and characteristics with treatment options and their outcomes. Results of such process may lead to stratification of patients to match them with tailored treatments. Several drug candidates identified using these methods are currently under investigation in clinical trials. An example is a mineralocorticoid receptor agonists in type 2 diabetes, particularly with mutation in NR3C2 gene to prevent renal failure, or interleukin-6 receptor agonists in amyotrophic lateral sclerosis [126]. As we integrate more advanced AI-based tools into data-driven medical practice, incorporating individual patient characteristics into treatment decision-making, such as drug choice or potential repositioning, may become an integral part of clinical routine.

This approach has been applied into a tool called mediKanren that can be utilized to aid clinicians working with rare diseases to process the significantly changed genes in each case. MediKanren works as a reasoning engine over knowledge graphs [127]. The graph was typically transformed into the database, which can be used for query search. MediKanren uses primarily structured data; however, as long as the unstructured data is implemented in the knowledge graph, it can also be incorporated into the analysis. The clinician evaluates genes or gene variants as potentially beneficial, for which MediKanren seeks potential drug candidates. While this tool does not provide any score, it gives the clinical associations to manually review and assign treatment. For example, this pipeline identified levocarnitine as a potential treatment for patients with a variant of the TMLHE gene, who suffered from seizures, speech impairment, and impaired motor function. The mutation this patient was carrying resulted in a decrease in carnitine levels [128]. MediKanren was used to search for a drug to increase carnitine, which included levocarnitine, an FDA-approved supplement. This resulted in clinical improvement of the patient [127]. The tool also identified celecoxib as a potentially effective treatment for a missense mutation in the RHOBTB2 gene (linked to reduced dendritic development) [127]. This example shows that combining AI-based tools with clinical expertise can significantly help patients with specific genetic traits, introducing a few steps into genome-tailored therapies and personalized medicine.

## Challenges and future outlook

Although AI holds significant promise in drug repurposing, several challenges must be surmounted to fully capitalize on its potential. One major issue is the presence of conflicting information across extensive datasets. The vast and varied nature of biomedical data often lead to inconsistencies and divergent outcomes, complicating data analysis and interpretation. Additionally, a heavy reliance on textual data may fail to adequately capture the complexities of biological systems, thereby limiting the effectiveness of AI models and negatively impacting their predictive accuracy [129, 130]. Another essential challenge is the fragmented status of medical records. The absence of standardized and interoperable data systems hinders the seamless integration of diverse datasets, which are vital for thorough analysis and sound decision-making. Addressing these challenges requires the development of robust data integration frameworks, enhancing data curation practices, and implementing advanced AI models capable of effectively managing complex and heterogeneous data environments [129, 131, 132].

Quantum computers hold transformative potential for AI-driven drug repurposing, offering exponential increases in processing speed to screen vast chemical libraries and manage the scale and complexity of datasets. By leveraging quantum processors, these systems can represent and process high-dimensional molecular data more compactly and in parallel than classical computers [133]. Early hybrid quantum-classical pipelines demonstrate this potential by offloading resource-intensive tasks, like molecular energy estimation to quantum hardware, while handling large-scale data management on classical clusters. Quantum processors encode high-dimensional molecular descriptors, like atomic properties or bond configurations into quantum bit (qubit) amplitudes, compressing data representations and alleviating memory demands inherent to classical systems. This approach has proven effective in generative chemistry workflows on D-Wave hardware [133]. Hybrid systems also parallelize computations that would overwhelm classical GPUs, accelerating generative design tasks such as proposing novel scaffolds. Early results show orders-of-magnitude reductions in exploration time for focused chemical libraries [134].

To ensure responsible innovation, emerging quantum-AI architectures emphasize explainability and ethical modelling, integrating fairness-aware pipelines and regulatory traceability into biomedical workflows [135]. These advancements ensure that insights generated by quantum systems remain trustworthy and clinically actionable—a vital requirement for regulatory acceptance. Quantum computing offers a transformative solution for integrating and analysing vast biomedical datasets. Addressing classical limitations in memory and computational throughput enables efficient, interpretable, and ethical AI-driven discovery, making it a critical enabler for unlocking actionable insights from large-scale, complex biomedical and clinical information systems.

Despite rapid advances in AI and computational repurposing strategies, regulatory infrastructure has lagged behind technological innovation. The regulatory agencies, the U.S. Food and Drug Administration (FDA) and the European Medicines Agency (EMA), have begun acknowledging AI’s role in drug development. However, comprehensive, harmonized guidelines are still lacking. This regulatory uncertainty presents a significant barrier to clinical translation and commercial deployment [136–138].

To address these challenges, future frameworks must establish clear standards for AI model transparency, validation, and reproducibility. This includes:

- **Model interpretability**: AI systems used in repurposing must provide clinically understandable reasoning behind predictions [139, 140].
- **Audit trails**: Algorithms must produce traceable outputs with sufficient documentation of data sources, training parameters, and versioning history [141, 142].
- **Bias detection**: Systems should be evaluated for demographic, disease, or data-source bias, particularly in underrepresented populations or rare diseases [143, 144].
- **Pre-approval submissions**: Like pharmacokinetic or toxicity studies, AI models may require regulatory pre-submission with predefined endpoints and validation benchmarks [145, 146].

Ultimately, regulatory acceptance will depend on performance metrics and AI workflows’ ethical and technical robustness [138]. Multistakeholder collaboration, including input from regulators, technologists, clinicians, and ethicists, will be crucial to shaping a regulatory environment that fosters innovation while safeguarding patient welfare.

Data heterogeneity remains one of the most significant obstacles to scalable and reproducible AI applications in drug repurposing. Biomedical datasets often differ in structure, format, terminology, and quality, complicating efforts to train generalizable models. Additionally, proprietary restrictions and fragmented data silos further limit accessibility, especially in rare disease research where data scarcity is most pronounced [143, 147].

Future progress requires a global push toward unified, open-source biomedical data platforms that integrate multi-omics data (genomics, transcriptomics, proteomics), clinical records and trial data, molecular interaction databases, and biomedical literature.

**Figure 3:**
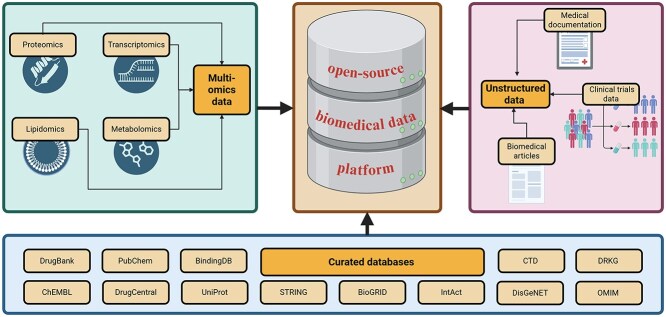
Components of a unified open-source biomedical data platform: A successful ecosystem must integrate diverse data modalities—multi-omics datasets (genomics transcriptomics proteomics) clinical records trial registries comprehensive biomedical literature as well as large curated repositories such as the Drug Repurposing Knowledge Graph (DRKG) ChEMBL and PubChem linked via common identifier mappings.

Standardization initiatives like FAIR (Findable, Accessible, Interoperable, and Reusable) data principles and harmonized metadata schemes will be instrumental. To ensure the long-term sustainability of large-scale data-driven research and the reuse of continuously generated data, a structured governance framework is essential to promote best practices among researchers and companies. Similarly, adoption of common technical frameworks for metadata and workflow would also make interoperability easier to achieve. Both data and software should be archived in online portals with sufficient metadata [148]. Integrating large, curated repositories, such as the Drug Repurposing Knowledge Graph (DRKG), ChEMBL, and PubChem, should serve as foundational nodes in a more comprehensive and interoperable data ecosystem [149, 150]. Ultimately, the success of unified platforms or adaptation of good practices still depends on sustained systemic investments in open infrastructure that makes them collaboratively accessible [148]. Figure 3 summarizes the components of a unified, open-source biomedical data platform.

Equally important is data democratization: providing access not only to academic researchers but also to under-resourced institutions, public health agencies, and international partners [151]. Such inclusivity will enhance algorithm robustness across diverse populations and support equity in therapeutic development [152].

## Conclusions

The future of AI-driven drug repurposing is promising, with several key directions poised to shape the field. One area of focus is the development of more sophisticated AI models that can better handle the complexities of biomedical data. This includes integrating multi-omics data, which combines genomic, proteomic, and metabolomic information to provide a more holistic view of disease mechanisms and drug interactions. Another important direction is the advancement of quantum computing technologies. Quantum computers offer the potential to increase processing speeds exponentially, enabling the analysis of vast chemical libraries and complex datasets. By leveraging quantum processors, researchers can overcome the limitations of classical computing, paving the way for more efficient and accurate drug discovery processes. Finally, there is a growing emphasis on ensuring AI’s ethical and responsible use in drug repurposing. This includes developing explainable AI models, which provide transparency and accountability in decision-making processes. By addressing these future directions, AI-driven drug repurposing can continue to evolve, offering new solutions to pressing medical challenges and improving patient outcomes.

**Key Points**

- Varied nature of biomedical data often leads to divergent outcomes in drug development.
- Reliance on textual data limits capturing complexities of biological systems, thereby limiting the effectiveness of AI models.
- Robust data integration frameworks enhance data curation practices in drug discovery and development.
- Advanced AI models are able of effectively manage complex and heterogeneous data environments.

## Data Availability

No original data were used in this review.
